# Diminished Exercise Capacity and Mitochondrial *bc1* Complex Deficiency in Tafazzin-Knockdown Mice

**DOI:** 10.3389/fphys.2013.00074

**Published:** 2013-04-17

**Authors:** Corey Powers, Yan Huang, Arnold Strauss, Zaza Khuchua

**Affiliations:** ^1^Division of Molecular Cardiovascular Biology, Cincinnati Children’s Medical CenterCincinnati, OH, USA

**Keywords:** Barth syndrome, tafazzin, complex-III, cardiolipins, exercise intolerance, mouse models

## Abstract

The phospholipid, cardiolipin, is essential for maintaining mitochondrial structure and optimal function. Cardiolipin-deficiency in humans, Barth syndrome, is characterized by exercise intolerance, dilated cardiomyopathy, neutropenia, and 3-methyl-glutaconic aciduria. The causative gene is the mitochondrial acyl-transferase, tafazzin, that is essential for remodeling acyl chains of cardiolipin. We sought to determine metabolic rates in tafazzin-deficient mice during resting and exercise, and investigate the impact of cardiolipin-deficiency on mitochondrial respiratory chain activities. Tafazzin-knockdown in mice markedly impaired oxygen consumption rates during an exercise, without any significant effect on resting metabolic rates. CL-deficiency resulted in significant reduction of mitochondrial respiratory reserve capacity in neonatal cardiomyocytes that is likely to be caused by diminished activity of complex-III, which requires CL for its assembly and optimal activity. Our results may provide mechanistic insights of Barth syndrome pathogenesis.

## Introduction

Phospholipids are building blocks of biological membranes. The mitochondrial inner membrane contains a unique phospholipid – cardiolipin that constitutes about 20% of total phospholipids (Schlame et al., 2005). The predominant form of CL in mammals is tetra-linoleoyl cardiolipin, or L4CL, which contains 4 linoleic acyl side chains (C18:2, *n*−6) (Schlame et al., 1993). L4CL is especially enriched in mitochondria of cardiac and skeletal muscles. CL is essential for assembly of respiratory chain (RC) complexes and their optimal activities (Fry and Green, 1981; Zhang et al., 2002, 2005; Pfeiffer et al., 2003; Acehan et al., 2011).

One of the clues to the importance of L4CL in mitochondrial function was provided when it was recognized that L4CL is markedly deficient in the mitochondria of patients with Barth syndrome (BTHS) (MIM 302060) (Valianpour et al., 2002). BTHS is a rare X-linked recessive genetic disorder caused by mutations in tafazzin gene on the X-chromosome. Taz is a mitochondrial transacylase required for CL remodeling and formation of L4CL. BTHS is characterized by dilated cardiomyopathy, exercise intolerance, chronic fatigue, skeletal muscle weakness, and cyclic or intermittent neutropenia.

Tafazzin-knockdown (Taz-KD) creates a mouse model of BTHS that resulted in marked reduction of L4CL both in cardiac and skeletal muscles (Acehan et al., 2010; Soustek et al., 2010). Prenatal loss of taz-deficient embryos due to cardiac abnormalities was reported at E12.5–E14.5 (Phoon et al., 2012). Transmission electron microscopy revealed mitochondrial damage and excessive mitophagy in striated muscles, consistent with findings in human BTHS samples (Acehan et al., 2010; Soustek et al., 2010).

Currently it is unknown how cardiolipin-deficiency affects principal energy-producing systems, such as RC complexes, in mammalian mitochondria. In the current study, we examine the impact of CL-deficiency on exercise capacity, oxygen utilization, respiratory exchange ratio (RER), and energy expenditure at rest and during exercise in Taz-KD mice. In addition, Taz-KD drastically diminishes mitochondrial respiratory reserve capacity, which is caused by reduced activity of mitochondrial RC complex III in CL-depleted mitochondria.

## Materials and Methods

### Animal procedures

All animal studies were approved by our Institutional Animal Care and Use Committee. Animals were housed in micro-isolator cages with temperature-controlled conditions under a 14/10 h light/dark cycle with free access to drinking water and food. Taz-KD was induced by introduction of doxycycline, as described previously (Acehan et al., 2010). Genotyping was performed by PCR analysis of tail genomic DNA (Acehan et al., 2010). Only males were used in experiments.

### Exercise on treadmill and open-circuit calorimetry

Metabolic rates were measured at rest and during exercise as described by G. Faldt et al. (2004). The resting oxygen consumption and carbon dioxide production rates (VO_2_ and VCO_2_, respectively) were measured at 31°C (thermoneutrality) every 10 min using the Oxymax system (Columbus Instrument, Columbus, OH, USA) for 24 h and normalized to mouse body weight. Normalized VO_2_ and VCO_2_ values at cold (+5°C) were measured every 10 min during a 5 h-period. Measurements were performed in metabolic chambers without food, but with free access to water. Fresh air was delivered into chambers with an electric pump.

Mice were exercised on a sealed motorized treadmill that had adjustable speed and inclination and was equipped with an electric shock-delivering grid. Electric shock intensity was set to 1 mA. Fresh air was delivered with an electric pump. Gas samples from the treadmill chamber were collected every 30 s and analyzed by the Oxymax system for measurement of VO_2_ and VCO_2_. RER, also known as the respiratory quotient, was calculated as VO_2_/VCO_2_. Open-circuit calorimetry results were calculated using Clax software (Columbus Instrument, Columbus, OH, USA).

### Culture of cardiomyocytes and mitochondrial respiration measurement

All reagents were purchased from Sigma Aldrich (St. Louis, MO, USA), unless otherwise noted. Neonatal cardiac myocytes were isolated from the hearts of 1 day old WT and Taz-KD neonatal mice as previously described (Khuchua et al., 1998). Viable cells were counted with a hemocytometer and plated at 20,000–50,000 cells/well density on laminin-coated XF24 plates. Cells were cultured for 48–72 h in a CO_2_ incubator at 37°C. The cardiomyocytes genotype in each well was determined by PCR-genotyping of tail samples from the corresponding carcass. One hour before measurements on an XF24 extracellular flux analyzer (Seahorse Bioscience, Billerica, MA, USA), cells were removed from the CO_2_ incubator and placed at 37°C in normal atmosphere, and media was replaced with 500 μl FX assay media composed of 143 mM NaCl, 5.4 mM KCl, 0.8 mM MgSO_4_, 0.91 mM Na_2_HPO_4_, 2 mM glutamine, 2 mg/ml BSA, and 15 mg/L phenol red, pH 7.4. Stock solutions (X10) of oligomycin, FCCP, and rotenone were prepared in FX assay media and loaded into injection ports A, B, and C, respectively. Measurements were obtained at 37°C.

### Isolation of mitochondria

Mitochondria were isolated from fresh adult mouse cardiac muscle. Animals were euthanized using ketamine (100 mg/kg), and hearts were quickly excised and placed in ice-cold 0.9% NaCl solution. All procedures were performed on ice. Blood was removed by washing, and hearts were minced with fine scissors. Minced tissues were transferred into Dounce glass-Teflon homogenizer and washed three times with 4 ml of ice-cold mitochondria isolation media (MIM) composed of 0.3 M sucrose, 10 mM Tris-HCl (pH 7.4), and 1 mM EDTA. One ml of ice-cold 0.025% trypsin-EDTA solution (Gibco) was added to each sample, gently mixed and incubated on ice for 7 min. Trypsin activity was quenched by addition of 3 ml of MIM with 4 mg/ml BSA. Tissues were gently homogenized with a Teflon pestle using a motorized drive. Homogenates were centrifuged at 1000 × *g* for 5 min at 4°C. Supernatants were transferred into new tubes and mitochondria sedimented by centrifugation at 8000 × *g* for 10 min at 4°C. Mitochondrial pellets were washed three times with MIM containing 2 mg/ml BSA and finally resuspended in 50 μl MIM with 2 mg/ml BSA (Roche). Mitochondrial protein concentration was determined using the DC protein assay (BioRad). Mitochondrial preparations were aliquoted, frozen in liquid nitrogen, and stored at −80°C.

### Enzymatic activities

Activities of mitochondrial RC complexes were determined spectrophotometrically using Shimadzu UV-1700 spectrophotometer in digitonin-treated isolated mitochondria as described earlier (Barrientos, 2002; Wibom et al., 2003) and normalized to citrate synthase (CS) activities.

For pretreatment, 10 μl frozen mitochondrial pellet (approximately 200 μg) was resuspended in 90 μl of 20 mM Tris-HCl, 120 mM KCl, 2 mg/ml digitonin (Life Technologies), 0.5 mg/ml BSA, and kept on ice.

#### NADH: coenzyme Q (complex I)

C-I assay mixture contained following final composition: 10 μl pretreated mitochondria, 0.97 ml of 5 mM KH_2_PO_4_ (pH 7.5), 5 mM MgCl_2_, 0.24 mM CoQ1, 0.5 mM KCN, 1 mg/ml BSA, and 2.4 μg/ml antimycin A. Reaction was initiated with 0.02 mM NADH and reduction of absorbance at 340 nm was recorded with spectrophotometer before and after addition of rotenone (final concentration 2 μg/ml).

#### NADH: cytochrome c reductase (complex I + III)

Ten microliters of pretreated mitochondria were incubated for 5 min at 30°C in 0.98 ml of 5 mM KH_2_PO_4_ (pH 7.5), 5 mM MgCl_2_, 0.24 mM CoQ1, 0.5 mM KCN, 1 mg/ml BSA, 0.12 mM cytochrome *c* (oxidized form). Reaction was initiated with 0.02 mM NADH and increase of absorbance at 550 nm was recorded with spectrophotometer before and after addition of antimycin A (final concentration 2 μg/ml).

#### Cytochrome c oxidase (complex IV)

Non-enzymatic oxidation of cytochrome c was followed at 550 nm in 0.99 ml of 50 mM KH_2_PO_4_ (pH 7.5), 2 μg/ml rotenone, and 0.03 mM reduced cytochrome *c*. Reduced cytochrome *c* was prepared using ascorbate (Birch-Machin et al., 1994). Ten microliters of pretreated mitochondria were added to reaction buffer and enzyme-catalyzed cytochrome c oxidation was measured before and after addition of 0.20 mM KCN.

#### Mitochondria ATPse activity (Complex V)

C-V assay media containing 50 mM Tris (pH 8.0), 5 mg/ml BSA, 20 mM MgCl_2_, 50 mM KCl, 15 μM FCCP, 5 μM antimycin A, 10 mM phosphoenol pyruvate, 2.5 mM ATP, 2 U/ml of lactate dehydrogenase and pyruvate kinase, and 0.02 mM NADH. Reaction was initiated by adding 10 μl of pretreated mitochondria and reaction was followed by reduction of NADH absorbance at 340 nm before and after addition 2 μM of oligomycin.

#### Citrate synthase

Citrate synthase activity was measured at 412 nm. CS assay media contained 0.1 mM 5,5′-dithiobis (2-nitrobenzoic acid); 3-carboxy-4-nitrophenyl disulfide (DTNB), 0.25% Triton X-100, 0.5 mM oxaloacetate, 0.31 mM acetyl CoA, 50 mM Tris-HCl, pH 8.0. CS activity was calculated by increasing absorbance at 412 nm using extinction coefficient for TNB 13.6 mM^−1^ × cm^−1^.

### Statistical analysis

Differences between groups were assessed for significance by unpaired Student’s *t*-test with the assumption of equal variances. Results were considered statistically significant if the *P* value was <0.05. Results are expressed as arithmetic means ± SEM. Statistical calculations were performed using the Prism program (GraphPad Software, San Diego, CA, USA).

## Results

### Energy expenditure and RER during rest and forced exercise on treadmill

Impaired ability to withstand physiological and environmental stressors, such as physical exercise or cold exposure, is a common feature for many mitochondrial myopathies. Exercise intolerance is one of the main clinical manifestations of BTHS in humans and has been linked to reduced ability of CL-deficient mitochondria to extract and utilize oxygen from blood (Spencer et al., 2011). Cold-intolerance has been reported in mouse models of fatty acid oxidation deficiency (Exil et al., 2006) and uncoupling protein knockout mice (Enerback et al., 1997). We investigated whether the mitochondrial abnormalities in striated muscles in Taz-KD model affect the ability of mice to withstand physiological and environmental stressors, such as physical exercise and cold environment. We were particularly interested if intolerance to stressors became manifest prior to the cardiac phenotype that becomes apparent at 7–8 months of age. Therefore, for exercise and indirect calorimetry experiments, we selected 4–5 month old WT and Taz-deficient male littermates. Metabolic indices in WT and Taz-KD mice were analyzed at rest and during exercise using an open-circuit indirect calorimetry.

**Figure 1:**
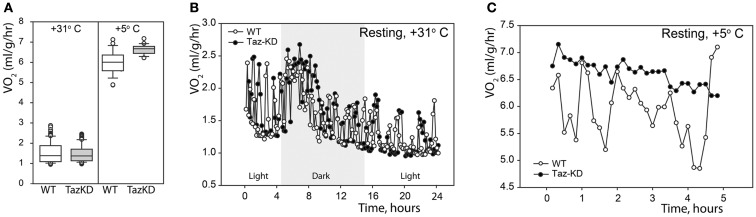
**Resting physiological indices of Taz-KD and WT mice at thermoneutrality (+31° C) and in the cold (+5° C). (A)** Whisker box plot of normalized oxygen consumption rates of WT (white) and Taz-KD (gray) mice at +31 and +5° C. **(B)** Representative plot of normalized oxygen consumption of WT and Taz-KD mice during a 24-h period at +31° C. Dark and light cycles are shown. **(C)** Representative plot of normalized oxygen consumption of WT and Taz-KD mice during a 5-h period at +5°C.

First, we analyzed the resting metabolic rate at thermoneutrality (+31°C) and in the cold (+5°C). Mice were placed in the temperature-controlled metabolic chambers without food but with free access to water. Oxygen consumption and CO_2_ production rates were measured every 10 min in small gas samples taken from the chamber using an online open-circuit indirect calorimetry system. Analysis showed that there were no differences in the resting oxygen consumption rates (VO_2_) between Taz-KD and WT groups at +31°C. Exposure to cold significantly increased VO_2_ in both experimental groups, but, again, values did not differ between WT and Taz-KD mice (Figure 1A). These results demonstrate that thermogenic capacity is not affected by CL-deficiency and that Taz-KD mice can adjust well and tolerate a cold environment. Monitoring of oxygen consumption during a 24-h-period at +31°C revealed that VO_2_ values follow periodic oscillating patterns, as shown on Figure 1B. Similarly, oscillating patterns of VO_2_ were observed in WT mice, when placed in cold environment (Figure 1C). In contrast, Taz-KD mice VO_2_ oscillation amplitudes were significantly less than those for WT controls.

Next, we subjected mice to forced exercise on a treadmill. After initial resting on the treadmill for 30 min to acclimate the animals, the test was started with a 10% incline and 5 m/min speed. Speed was increased step-wise by 5 m/min every 5 min to a final speed of 25 m/min. Thus, the duration of an exercise session was 36.8 min and the distance traveled was 507.4 m. Taz-KD mice repeatedly failed to stay on the belt at 15 m/min and 10% inclination, and none of the Taz-KD mice were able to sustain running when the treadmill speed more than 20 m/min. In contrast, WT control mice had no difficulty maintaining exercise at this speed. Because Taz-KD mice could not sustain exercise on the treadmill at 10% incline, we reduced the incline to 5%, and mice of both experimental groups were able to tolerate this workload during the entire 36.8 min session.

**Figure 2:**
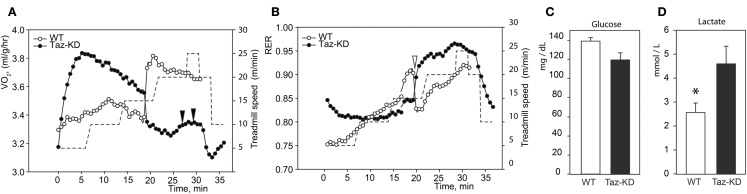
**Representative recordings of physiological parameters of Taz-KD and WT mice during forced exercise on a treadmill. (A,B)** O_2_ consumption rate and respiration exchange ratio (RER) were monitored continuously during the entire session of forced exercise. Taz-KD mice repeatedly failed to stay on treadmill [black arrowheads on **(A)**], while WT controls remained on the treadmill belt during the entire test. Sudden drops of RER values were present in all tested WT mice [white arrowhead on **(B)**] and were totally absent or substantially blunted in Taz-KD mice. Treadmill speed is shown with dashed line [right hand axis of **(A,B)**]. Blood glucose **(C)** and lactate **(D)** levels were measured in WT and Taz-KD mice immediately after completion of exercise testing (*n* = *4*). * P < *0.05*.

Indirect calorimetric analysis revealed that VO_2_ values sharply rose with increased workload in Taz-KD animals, while those for WT mice remained relatively steady (Figure 2A). Paradoxically, with further increases of running speed, VO_2_ values for Taz-KD mice declined, but WT mice VO_2_ continued to rise with increasing workload (Figure 2A). Analysis revealed a sudden drop of RER values during exercise with increasing speed (Figure 2B). This drop of RER values was common for all tested WT mice. Surprisingly this phenomenon is either absolutely absent or significantly subtler in Taz-KD mice (Figure 2B). Blood glucose and lactate were analyzed at the end of exercise sessions. Blood glucose levels were not significantly different between WT and Taz-KD groups (Figure 2C); however, lactate was significantly elevated in blood samples of Taz-KD mice compared to WT controls (Figure 2D), indicative of impaired aerobic energy metabolism in Taz-deficient mice.

### Metabolic profiling of neonatal cardiomyocytes

**Figure 3:**
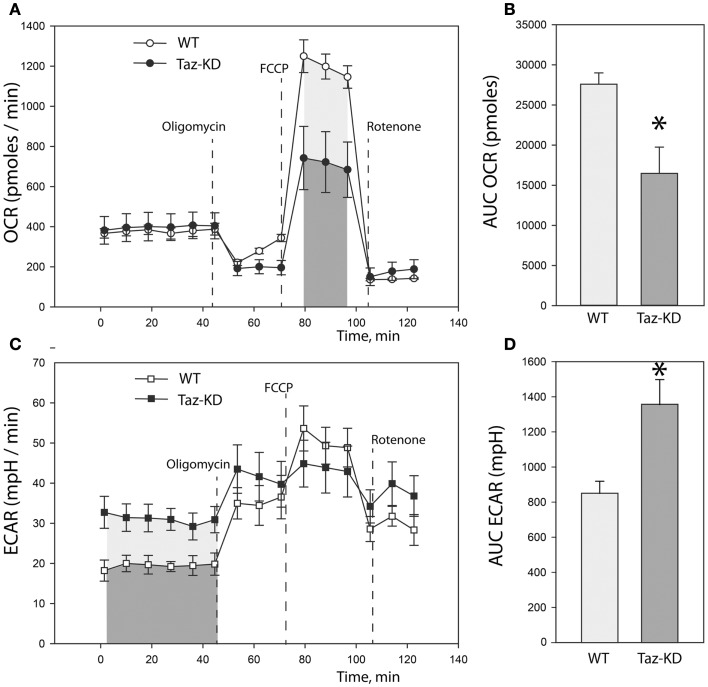
**Metabolic profiling of neonatal cardiomyocytes from WT and Taz-KD mice. (A)** Basal and stimulated mitochondrial oxygen consumption rates (OCR) in cultured cardiomyocytes. OCR traces are expressed as pmol O_2_ per min in WT and Taz-KD cardiomyocytes and normalized to cell number. Vertical dashed lines indicate the times of addition of oligomycin (2 μM), FCCP (3.7 μM), and rotenone (2 μM). **(B)** Total oxygen consumption (reserve capacity) is significantly lower in Taz-KD cardiomyocytes compared with the WT controls (*p < *0.007*). Mitochondrial reserve capacity was determined by calculating the total area under the curve (AUC) of FCCP-stimulated respiration trace [shaded areas on **(A)**]. **(C)** Extracellular acidification rates (ECAR) of WT and Taz-KD neonatal cardiomyocytes. ECAR rates, expressed as mpH per min in WT and Taz-KD cardiomyocytes and normalized to cell numbers. **(D)** ECAR values at basal conditions were significantly higher for Taz-KD cardiomyocytes (*p < *0.002*), consistent with an increase reliance of Taz-KD cells on glycolysis. Extracellular acidification values were determined by calculating AUC of ECAR tracing at basal metabolic state [shaded areas on **(C)**]. The differences in means in **(B,D)** were assessed by Tukey’s post-hoc test.

Previously, we demonstrated that L4CL content is greatly reduced in Taz-deficient cardiac mitochondria (Acehan et al., 2010). Reduction of L4CL is likely to affect enzymatic activities of the electron transport chain. To further characterize defects of mitochondrial metabolism within the context of intact cells, we prepared primary cultures of neonatal cardiomyocytes from WT and Taz-KD mice. We analyzed mitochondrial oxygen consumption rate (OCR) and extracellular acidification rate (ECAR) in cultured neonatal cardiomyocytes using the XF24 Seahorse bioanalyzer (Figure 3). First, basal respiration rates of cardiomyocytes were determined (Figure 3A). Following the measurements of basal OCR values, oligomycin, a complex V inhibitor, was introduced into the respiration media to distinguish ATP-linked respiration from the proton leak. As shown on Figure 3A, basal respiration rates and proton leak values were not different between WT and taz-deficient cardiomyocytes. Following oligomycin injection, maximal, or uncoupled respiration rate was determined by injecting wells with 3.8 μM FCCP. Taz-KD cardiomyocytes exhibited approximately 40% lower maximal respiration values than WT controls; presumably signifying reduced activities of ETC complexes in CL-deficient mitochondria.

Coincident with this decrease in mitochondrial oxygen consumption, we noted that the basal ECAR in Taz-KD cardiomyocytes was higher than in WT controls, consistent with an increased reliance on glycolysis (Figures 3C,D). This metabolic shift away from aerobic respiration and toward cytosolic glycolysis in CL-deficient cardiomyocytes probably indicates a compensatory remodeling of cellular metabolism in order to maintain cellular energy homeostasis in the setting of dysfunctional mitochondria.

### Measurement of activities of individual mitochondrial RC complexes

Reduction of maximal respiration rates in Taz-KD cells suggest that the mitochondrial oxidative phosphorylation system is damaged and that activities of RC complexes are compromised in CL-depleted mitochondria. However, it is not clear which RC complex is most affected by CL-insufficiency.

We examined activities of individual mitochondrial complexes in cardiac mitochondria. Mitochondrial fractions were isolated from doxycyline-fed WT and Taz-KD mice of 3–4 months of age. Mitochondria were solubilized with digitonin. Enzymatic activities of complex I (NADH-dehydrogenase), complex I–III segment, complex IV (cytochrome c oxidase), and complex V (mitochondrial ATPase) were measured spectrophotometrically. Activities of mitochondrial RC complexes were normalized to CS activities for each mitochondrial preparation.

**Figure 4:**
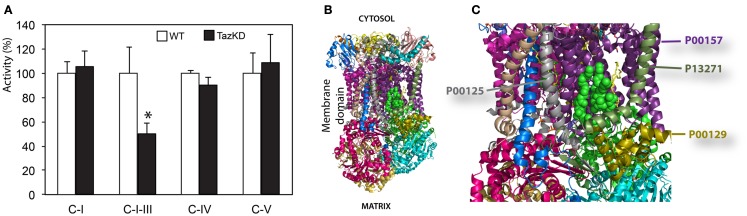
**Impaired complex-III activity in CL-deficient mitochondria. (A)** Activities of individual respiratory chain complexes in isolated cardiac mitochondria of WT and Taz-KD mice. **(B)** The structure of bovine *bc1* complex (C-III) with bound phospholipid molecules (PDB entry 2AO6). Different polypeptides are shown with various colors. Sides facing cytosol and mitochondrial matrix sides are shown. **(C)** magnified view of phospholipids moiety in mitochondrial *bc1* complex. Two cardiolipin molecules are illustrated with green balls. Phosphatidyl-ethanolamine molecules are shown as yellow sticks. Polypeptides in close proximity to phospholipid molecules are shown: cytochrome c1 (P00125, gray), cytochrome b (P00157, purple), subunit 8, or ubiquinone-binding protein QP-C (P13271, dark olive) and subunit 7 (P00129, gold).

We found that rotenone-sensitive complex-I, cyanide-sensitive complex IV, and oligomycin-sensitive complex V activities were not significantly affected by CL-deficiency in Taz-KD cardiac mitochondria. In contrast, activity of RC segment complex I–III (cytochrome *c* oxidoreductase or C-I–C-III) was reduced in Taz-KD mitochondria by 40% (Table 1, Figure 4A), suggesting that mitochondrial complex III activity is diminished in CL-deficient mitochondria.

**Table 1:** **Enzymatic activities of mitochondrial respiratory chain complexes in WT and Taz-KD mitochondria**.

	WT	Taz-KD
Complex I	0.215 ± 0.020 (n = 6)	0.227 ± 0.028 (n = 4)	ns
Complex I – III	1.289 ± 0.393 (n = 5)	0.574 ± 0.105 (n = 5)	P < 0.05
Complex IV	4.382 ± 0.085 (n = 6)	3.960 ± 0.269 (n = 4)	ns
Complex V	0.357 ± 0.059 (n = 3)	0.388 ± 0.084 (n = 3)	ns

*Values are normalized to citrate synthase activities and presented as a mean ± SEM (n, number of assays; ns, not statistically significant)*.

## Discussion

In the present study, we investigated mechanisms of exercise intolerance in a mouse model of BTHS. Exercise intolerance has been recognized as one of the hallmarks of human BTHS. It has been suggested that exercise intolerance in BTHS patients is caused by diminished extraction/utilization of oxygen by skeletal muscle and impaired cardiac contractile reserve (Spencer et al., 2011). However, molecular mechanisms underlying the effects of CL-deficiency on mitochondrial function in muscle cells remain unknown.

We demonstrated that CL-deficiency in Taz-deficient mice had no significant effect on resting metabolism in either warm or cold environments. Both WT and Taz-KD mice equally responded to cold stress by robustly increasing oxygen consumption. Cold-intolerance has been described in genetic models with defects in mitochondrial energy-producing systems. However, our results demonstrated that Taz-KD mice did not show any signs of distress while exposed to cold environment (+5°C) for more than 5 h. More detailed analysis of metabolic parameters in a warm environment revealed that OCR of WT control mice follow periodic oscillating patterns, perhaps reflecting murine circadian activities (Figure 1B). Similar oscillating patterns of O_2_ consumption rates were observed in WT mice when placed in cold environment (Figure 1C). In contrast, in Taz-KD mice VO_2_ oscillation was either completely absent or amplitudes were significantly reduced as compared to WT controls. These results may be indicative that mechanisms of dynamic regulation of whole-body metabolism are affected by CL-deficiency in mitochondria.

When subjected to forced exercise on treadmill, Taz-KD mice performed far more poorly than WT littermates. Taz-KD mice were not able to sustain a high-intensity exercise (20 m/min at 10% inclining) even for 30 s, while WT control littermates remained on the belt at this level of intense exercise. Open-circuit calorimetry during aerobic exercise on the treadmill revealed that, in response to step-wise increasing intensity of workload, mice progressively move metabolic reliance from mixed substrates (fat, carbohydrates, and amino acids) toward the carbohydrates. This shift in fuel preference is reflected as a gradual increase of RER values from 0.75 to 0.95 or higher with increasing workload. With further increases in running speed, RER values for Taz-KD mice sharply dropped, which indicates that WT mice dynamically shifted their reliance on metabolic substrates from predominantly carbohydrates (RER ≥ 0.9) to mixed substrates (RER ≤ 0.8). This switch, which was common for all tested WT mice, can be related to the “second wind” phenomenon in athletes (Bank and Chance, 1994). Surprisingly, this phenomenon is either absolutely absent or significantly reduced in Taz-KD mice. The “second wind” phenomenon is affected in humans with various inborn metabolic defects (Haller and Vissing, 2002, 2004; Vissing et al., 2005). Absence of a “second wind” may be further evidence that mechanisms of dynamic regulation of metabolism in response to energy demands are deficient in Taz-KD mice. Open calorimetry results are in good agreement with recently published data suggesting that BTHS patients have difficulties extracting oxygen from blood and/or utilizing it when performing an exercise on cycle ergometer (Spencer et al., 2011).

Cardiolipin molecules are associated with all RC complexes. Cardiolipin is essential for interactions of RC complexes and assembly of supercomplexes (Zhang et al., 2005). Metabolic profiling of neonatal cardiomyocytes revealed that CL-deficiency had no apparent effect on basal oxygen consumption level; however, maximal uncoupled respiration rate was markedly reduced in Taz-KD cardiomyocytes compared to WT controls. Reduction of maximal respiration rate in Taz-KD cells can be caused by diminished activity of mitochondrial complex-III in CL-depleted mitochondria. Mitochondrial complex III (C-III), also called cytochrome *bc1* complex, is composed of 11 subunits (PDB: 2A06), of which of only one, cytochrome *b*, is encoded by the mitochondrial genome. C-III catalyzes the transfer of electrons from reduced coenzyme Q to cytochrome c, with a concomitant translocation of protons across the inner mitochondrial membrane (Benit et al., 2009; Wenz et al., 2009; Gil Borlado et al., 2010). Bovine cardiac C-III contains four structurally incorporated CL molecules (Huang et al., 2005). The head groups of one cardiolipin molecule bind at the interface of cytochrome b (P00157) and subunit 7 (P00129) and might be important for the structural integrity of the complex (Figures 4B,C). Cardiolipin is essential for super-complex formation between C-III and C-IV in yeast mitochondria (Zhang et al., 2005). CL molecules may also participate in forming the environment necessary to promote substrate diffusion from the membrane to the active site and/or substrate exchange between sites of quinine/quinol catalysis within the complex (Palsdottir and Hunte, 2004).

Mitochondrial DNA (mtDNA) mutations in cytochrome *b (MT-CYB)* gene constitute a major cause of complex III deficiency and underlie a wide range of neuromuscular disorders (Gil Borlado et al., 2010), with exercise intolerance as a major symptom (Andreu et al., 1999). Ischemia-reperfusion injury in rat heart causes reduction of C-III with concomitant decrease of CL content in mitochondria (Petrosillo et al., 2003).

Reduced C-III activity perhaps may not be the only factor that results in diminished maximal respiration in Taz-KD cells. Other factors, such as limited lateral diffusion of electron-transporting carriers and destabilization of RC supercomplexes in CL-depleted mitochondria, cannot be entirely excluded. It has been demonstrated that CL is required for the assembly of supramolecular complexes of complex-V (ATP synthase complex) (Acehan et al., 2011). In a recent publication (Kiebish et al., 2013) it has been shown that Taz-knockdown affects C-III activity in cardiac mitochondria without any effects on C-I, C-II, and C-IV activities. However, in contrast with our studies the authors observed a relatively small, but statistically significant deficiency in C-V activity in Taz-KD cardiac mitochondria. This discrepancy with our results may be explained by differences in temperatures of C-V assays. In our experiments, we measured C-V activity at 30°C, while Kiebish et al. at 37°C. It is plausible that C-V deficiency is not perceptible at 30°C, but manifests at higher temperature. Moreover, in our C-V assay, we measured ATP hydrolysis rate, the reverse reaction to ATP synthesis. It is possible that the presence of CL is critical for the ATP synthase reaction, but has less effect on ATP hydrolysis.

Possible enhanced production of reactive oxygen species (ROS) in CL-deficient mitochondria and increased ROS-mediated damage of mtDNA may be additional pathogenic factors in developing of phenotype in Taz-KD mice and BTHS patients.

In summary, we report exercise intolerance in Taz-KD mice with markedly impaired oxygen utilization capability at high workload, but without apparent deficiencies at rest. CL-deficiency resulted in significant reductions of maximal uncoupled mitochondrial respiration rate, or mitochondrial reserve in Taz-KD neonatal cardiomyocytes. Reduction of mitochondrial reserve in Taz-KD cardiomyocytes is likely caused by diminished activity of complex-III, which requires CL for its assembly and optimal activity. Our results provide a mechanistic insight of pathogenesis of BTHS and may be useful for designing potential therapeutic interventions, such as increasing the mitochondrial RC efficiency by supplementations with vitamins K_2_ and C (Argov et al., 1986; Vos et al., 2012).

## Conflict of Interest Statement

The authors declare that the research was conducted in the absence of any commercial or financial relationships that could be construed as a potential conflict of interest.

## References

[B1] Acehan D. Malhotra A. Xu Y. Ren M. Stokes D. L. Schlame M. (2011). Cardiolipin affects the supramolecular organization of ATP synthase in mitochondria. Biophys. J. 100, 2184–2192 10.1016/j.bpj.2011.03.031 21539786 PMC3150712

[B2] Acehan D. Vaz F. Houtkooper R. H. James J. Moore V. Tokunaga C. (2010). Cardiac and skeletal muscle defects in a mouse model of human Barth syndrome. J. Biol. Chem. 286, 899–908 10.1074/jbc.M110.171439 21068380 PMC3020775

[B3] Andreu A. L. Hanna M. G. Reichmann H. Bruno C. Penn A. S. Tanji K. (1999). Exercise intolerance due to mutations in the cytochrome b gene of mitochondrial DNA. N. Engl. J. Med. 341, 1037–1044 10.1056/NEJM199909303411404 10502593

[B4] Argov Z. Bank W. J. Maris J. Eleff S. Kennaway N. G. Olson R. E. (1986). Treatment of mitochondrial myopathy due to complex III deficiency with vitamins K3 and C: A 31P-NMR follow-up study. Ann. Neurol. 19, 598–602 10.1002/ana.410190615 3014998

[B5] Bank W. Chance B. (1994). An oxidative defect in metabolic myopathies: diagnosis by noninvasive tissue oximetry. Ann. Neurol. 36, 830–837 10.1002/ana.410360606 7998768

[B6] Barrientos A. (2002). In vivo and in organello assessment of OXPHOS activities. Methods 26, 307–316 10.1016/S1046-2023(02)00036-1 12054921

[B7] Benit P. Lebon S. Rustin P. (2009). Respiratory-chain diseases related to complex III deficiency. Biochim. Biophys. Acta 1793, 181–185 10.1016/j.bbamcr.2008.06.004 18601960

[B8] Birch-Machin M. A. Briggs H. L. Saborido A. A. Bindoff L. A. Turnbull D. M. (1994). An evaluation of the measurement of the activities of complexes I-IV in the respiratory chain of human skeletal muscle mitochondria. Biochem. Med. Metab. Biol. 51, 35–42 10.1006/bmmb.1994.1004 8192914

[B9] Enerback S. Jacobsson A. Simpson E. M. Guerra C. Yamashita H. Harper M. E. (1997). Mice lacking mitochondrial uncoupling protein are cold-sensitive but not obese. Nature 387, 90–94 10.1038/387090a0 9139827

[B10] Exil V. J. Gardner C. D. Rottman J. N. Sims H. Bartelds B. Khuchua Z. (2006). Abnormal mitochondrial bioenergetics and heart rate dysfunction in mice lacking very-long-chain acyl-CoA dehydrogenase. Am. J. Physiol. 290, H1289–H1297 10.1152/ajpheart.00811.2005 16199475

[B11] Faldt J. Wernstedt I. Fitzgerald S. M. Wallenius K. Bergstrom G. Jansson J. O. (2004). Reduced exercise endurance in interleukin-6-deficient mice. Endocrinology 145, 2680–2686 10.1210/en.2003-1319 14988384

[B12] Fry M. Green D. E. (1981). Cardiolipin requirement for electron transfer in complex I and III of the mitochondrial respiratory chain. J. Biol. Chem. 256, 1874–1880 6257690

[B13] Gil Borlado M. C. Moreno Lastres D. Gonzalez Hoyuela M. Moran M. Blazquez A. Pello R. (2010). Impact of the mitochondrial genetic background in complex III deficiency. PLoS ONE 5:e12801 10.1371/journal.pone.0012801 20862300 PMC2941448

[B14] Haller R. G. Vissing J. (2002). Spontaneous “second wind” and glucose-induced second “second wind” in McArdle disease: oxidative mechanisms. Arch. Neurol. 59, 1395–1402 10.1001/archneur.59.9.1395 12223025

[B15] Haller R. G. Vissing J. (2004). No spontaneous second wind in muscle phosphofructokinase deficiency. Neurology 62, 82–86 10.1212/WNL.62.1.82 14718702

[B16] Huang L. S. Cobessi D. Tung E. Y. Berry E. A. (2005). Binding of the respiratory chain inhibitor antimycin to the mitochondrial bc1 complex: a new crystal structure reveals an altered intramolecular hydrogen-bonding pattern. J. Mol. Biol. 351, 573–597 10.1016/j.jmb.2005.05.053 16024040 PMC1482829

[B17] Khuchua Z. A. Qin W. Boero J. Cheng J. Payne R. M. Saks V. A. (1998). Octamer formation and coupling of cardiac sarcomeric mitochondrial creatine kinase are mediated by charged N-terminal residues. J. Biol. Chem. 273, 22990–22996 10.1074/jbc.273.36.22990 9722522

[B18] Kiebish M. A. Yang K. Liu X. Mancuso D. J. Guan S. Zhao Z. (2013). Dysfunctional cardiac mitochondrial bioenergetic, lipidomic, and signaling in a murine model of Barth syndrome. J. Lipid Res. [Epub ahead of print]. 23410936 10.1194/jlr.M034728 PMC3622326

[B19] Palsdottir H. Hunte C. (2004). Lipids in membrane protein structures. Biochim. Biophys. Acta 1666, 2–18 10.1016/j.bbamem.2004.06.012 15519305

[B20] Petrosillo G. Ruggiero F. M. Di Venosa N. Paradies G. (2003). Decreased complex III activity in mitochondria isolated from rat heart subjected to ischemia and reperfusion: role of reactive oxygen species and cardiolipin. FASEB J. 17, 714–716 10.1096/fj.03-0012com 12586737

[B21] Pfeiffer K. Gohil V. Stuart R. A. Hunte C. Brandt U. Greenberg M. L. (2003). Cardiolipin stabilizes respiratory chain supercomplexes. J. Biol. Chem. 278, 52873–52880 [Epub October 5, 2003 52815]. 10.1074/jbc.M307540200 14561769

[B22] Phoon C. K. Acehan D. Schlame M. Stokes D. Edelman-Novemsky I. Yu D. (2012). Tafazzin knockdown in mice leads to a developmental cardiomyopathy with early diastolic dysfunction preceding myocardial noncompaction. J. Am. Heart Assoc. 10.1161/JAHA.111.000455 PMC3487377 23130124

[B23] Schlame M. Brody S. Hostetler K. Y. (1993). Mitochondrial cardiolipin in diverse eukaryotes. Comparison of biosynthetic reactions and molecular acyl species. Eur. J. Biochem. 212, 727–735 10.1111/j.1432-1033.1993.tb17711.x 8385010

[B24] Schlame M. Ren M. Xu Y. Greenberg M. L. Haller I. (2005). Molecular symmetry in mitochondrial cardiolipins. Chem. Phys. Lipids 138, 38–49 10.1016/j.chemphyslip.2005.08.002 16226238

[B25] Soustek M. S. Falk D. Mah C. Toth M. Schlame M. Lewin A. (2010). Characterization of a transgenic shRNA induced murine model of tafazzin deficiency. Hum. Gene Ther. 22, 865–871 10.1089/hum.2010.199 21091282 PMC3166794

[B26] Spencer C. T. Byrne B. J. Bryant R. M. Margossian R. Maisenbacher M. Breitenger P. (2011). Impaired cardiac reserve and severely diminished skeletal muscle O2 utilization mediate exercise intolerance in Barth syndrome. Am. J. Physiol. 301, H2122–H2129 10.1152/ajpheart.00479.2010 21873497

[B27] Valianpour F. Wanders R. J. Overmars H. Vreken P. Van Gennip A. H. Baas F. (2002). Cardiolipin deficiency in X-linked cardioskeletal myopathy and neutropenia (Barth syndrome, MIM 302060): a study in cultured skin fibroblasts. J. Pediatr. 141, 729–733 10.1067/mpd.2002.129174 12410207

[B28] Vissing J. Quistorff B. Haller R. G. (2005). Effect of fuels on exercise capacity in muscle phosphoglycerate mutase deficiency. Arch. Neurol. 62, 1440–1443 10.1001/archneur.62.9.1440 16157752

[B29] Vos M. Esposito G. Edirisinghe J. N. Vilain S. Haddad D. M. Slabbaert J. R. (2012). Vitamin K2 is a mitochondrial electron carrier that rescues pink1 deficiency. Science 336, 1306–1310 10.1126/science.1218632 22582012

[B30] Wenz T. Hielscher R. Hellwig P. Schagger H. Richers S. Hunte C. (2009). Role of phospholipids in respiratory cytochrome bc(1) complex catalysis and supercomplex formation. Biochim. Biophys. Acta 1787, 609–616 10.1016/j.bbabio.2009.02.012 19254687

[B31] Wibom R. Hagenfeldt L. von Dobeln U. (2003). Measurement of ATP production and respiratory chain enzyme activities in mitochondria isolated from small muscle biopsy samples. Anal. Biochem. 317, 139–151 10.1016/S0003-2697(03)00247-1 12470673

[B32] Zhang M. Mileykovskaya E. Dowhan W. (2002). Gluing the respiratory chain together. Cardiolipin is required for supercomplex formation in the inner mitochondrial membrane. J. Biol. Chem. 277, 43553–43556 10.1074/jbc.M202179200 12364341

[B33] Zhang M. Mileykovskaya E. Dowhan W. (2005). Cardiolipin is essential for organization of complexes III and IV into a supercomplex in intact yeast mitochondria. J. Biol. Chem. 280, 29403–29408 10.1074/jbc.M506275200 15972817 PMC4113954

